# Hybrid sequencing of the *Gynostemma pentaphyllum* transcriptome provides new insights into gypenoside biosynthesis

**DOI:** 10.1186/s12864-019-6000-y

**Published:** 2019-08-05

**Authors:** Tongtong Liang, Liqiu Zou, Sijie Sun, Xuejun Kuang, Jianhe Wei, Lizhi Wang, Ying Li, Chao Sun

**Affiliations:** 1 Institute of Medicinal Plant Development (IMPLAD), Chinese Academy of, Medical Sciences, No.151, Malianwa North Road, Haidian District, Beijing, 100193 China; 2 Tianjin University of Traditional Chinese Medicine, No.10, Poyanghu Road, Jinghai District, Tianjin, 301617 China

**Keywords:** *Gynostemma pentaphyllum*, Gypenosides, Biosynthesis, Iso-Seq, Transcriptome

## Abstract

**Background:**

Gypenosides are a group of triterpene saponins from *Gynostemma pentaphyllum* that are the same as or very similar to ginsenosides from the *Panax* species. Several enzymes involved in ginsenoside biosynthesis have been characterized, which provide important clues for elucidating the gypenoside biosynthetic pathway. We suppose that gypenosides and ginsenosides may have a similar biosynthetic mechanism and that the corresponding enzymes in the two pathways may have considerable similarity in their sequences. To further understand gypenoside biosynthesis, we sequenced the *G. pentaphyllum* transcriptome with a hybrid sequencing-based strategy and then determined the candidate genes involved in this pathway using phylogenetic tree construction and gene expression analysis.

**Results:**

Following the PacBio standard analysis pipeline, 66,046 polished consensus sequences were obtained, while Illumina data were assembled into 140,601 unigenes with Trinity software. Then, these output sequences from the two analytical routes were merged. After removing redundant data with CD-HIT software, a total of 140,157 final unigenes were obtained. After functional annotation, five 2,3-oxidosqualene cyclase genes, 145 cytochrome P450 genes and 254 UDP-glycosyltransferase genes were selected for the screening of genes involved in gypenoside biosynthesis. Using phylogenetic analysis, several genes were divided into the same subfamilies or closely related evolutionary branches with characterized enzymes involved in ginsenoside biosynthesis. Using real-time PCR technology, their expression patterns were investigated in different tissues and at different times after methyl jasmonate induction. Since the genes in the same biosynthetic pathway are generally coexpressed, we speculated that GpOSC1, GpCYP89, and GpUGT35 were the leading candidates for gypenoside biosynthesis. In addition, six GpWRKYs and one GpbHLH might play a possible role in regulating gypenoside biosynthesis.

**Conclusions:**

We developed a hybrid sequencing strategy to obtain longer length transcriptomes with increased accuracy, which will greatly contribute to downstream gene screening and characterization, thus improving our ability to elucidate secondary metabolite biosynthetic pathways. With this strategy, we found several candidate genes that may be involved in gypenoside biosynthesis, which laid an important foundation for the elucidation of this biosynthetic pathway, thus greatly contributing to further research in metabolic regulation, synthetic biology and molecular breeding in this species.

**Electronic supplementary material:**

The online version of this article (10.1186/s12864-019-6000-y) contains supplementary material, which is available to authorized users.

## Background

*Gynostemma pentaphyllum* (Thunb.) Makino, belonging to the Cucurbitaceae family, is a kind of perennial creeping herbaceous plant widely distributed in China, India, Japan, Korea and Southeast Asia [1, 2]. *G. pentaphyllum*, also known as “Southern Ginseng”, is a traditional Chinese medicinal herb and is reported to have an adaptogenic nature that enhances the “yin” and “yang” properties of the human body [3]. The main active components of *G. pentaphyllum* are gypenosides (triterpene saponins). To date, more than 200 different gypenosides have been isolated from *G. pentaphyllum* [4–7]. Modern pharmacological studies have shown that gypenosides contribute to antitumor, hypoglycemic, hypolipidemic, cardiovascular and cerebrovascular protection and immunoprotection [8–10]. Previous studies have shown that 25% of all gypenosides are similar to ginsenosides, especially Gyp III, IV, VIII and XII, which are exactly the same as Gin-Rb1, -Rb3, -Rd and -F2 [11]. The content of triterpene saponins in *G. pentaphyllum* is almost five times higher than that of *Panax ginseng*. In addition, *G. pentaphyllum* is much easier to culture and grows faster than *P. ginseng*. Therefore, *G. pentaphyllum* is a good alternative resource for gypenoside production and has attracted great interest worldwide [3, 12].

**Fig. 1:**
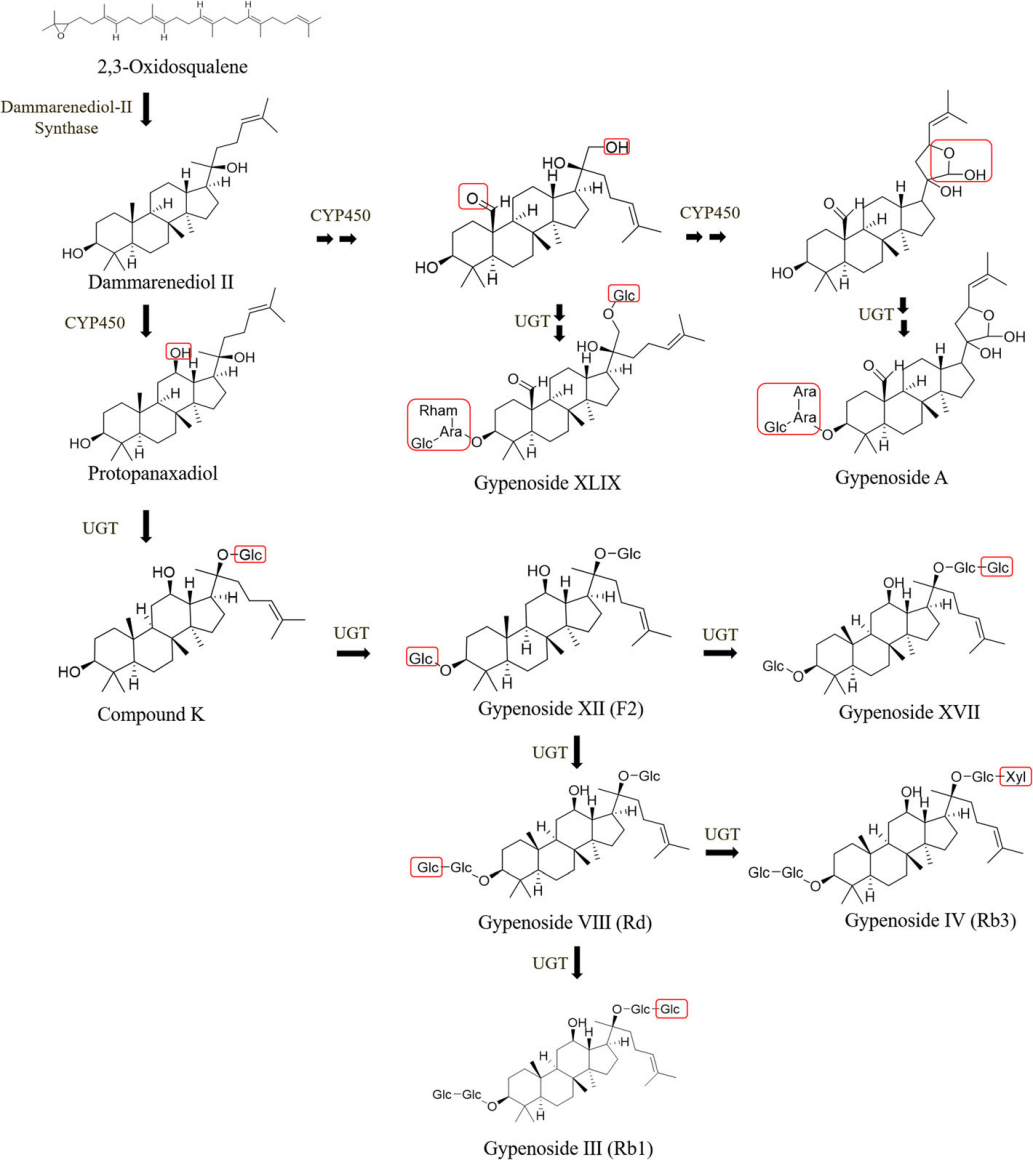
The putative gypenoside biosynthetic pathway

The gypenoside biosynthetic pathway can be divided into three parts: (1) Initial steps: the synthesis of isopentenyl diphosphate (IPP) or dimethylallyl pyrophosphate (DMAPP). In plants, IPP and DMAPP are common precursors for the synthesis of all terpenoids and sterols, which are usually synthesized by either the mevalonic acid (MVA) pathway in the cytoplasm or the methylerythritol phosphate (MEP) pathway in the plastid [13]. (2) Skeletal formation steps: the cyclization of 2,3-oxidosqualene. First, two molecules of IPP and one molecule of DMAPP are assembled to farnesyl pyrophosphate (FPP) by farnesyl pyrophosphate synthase (FPS). Then, two molecules of FPP are condensed to squalene by squalene synthase (SS), and squalene is further converted to 2,3-oxidosqualene by squalene epoxidase (SE). Finally, 2,3-oxidosqualene is cyclized into various triterpene skeletons by 2,3-oxidosqualene cyclases (OSCs). The cyclization of 2,3-oxidosqualene catalyzed by OSCs is the first branching point in triterpene synthesis and contributes to most of the skeleton diversity. (3) Modification steps: hydroxylation and glycosylation of the skeletons. Cytochrome P450s (CYP450s) are the major enzymes for skeletal hydroxylation, while UDP-glycosyltransferases (UGTs) are the major enzymes for skeletal glycosylation (Fig. 1) [14, 15]. As most gypenosides are tetracyclic triterpene dammarane saponins, dammarenediol-II synthase (DS) should be the most important OSC for gypenoside synthesis in *G. pentaphyllum*. In *P. ginseng*, DS has been characterized [16], and three other OSCs are also found, including β-amyrin synthase (BAS) [17, 18], lanosterol synthase (LS) [19] and cycloartenol synthase (CAS) [20]. Three CYP450s were identified from *P. ginseng*: CYP716A47 (protopanaxadiol synthase, PPDS), CYP716A53v2 (protopanaxatriol synthase, PPTS) and CYP716A52v2 (β-amyrin-28 oxidase). CYP716A47 catalyzed dammarenediol-II to form protopanaxadiol [21], CYP716A53v2 catalyzed protopanaxadiol to form protopanaxatriol [22], and CYP716A52v2 catalyzed β-amyrin to oleanolic acid [23]. Five UGTs were identified from *P. ginseng*, including UGTPg1, UGTPg100, UGTPg101, PgUGT94Q2 and PgUGT74AE2. PgUGT74AE2 can catalyze the glycosylation of C3-OH of protopanaxadiol and compound K to form ginsenoside Rh2 and F2, respectively [24]; PgUGT94Q2 can catalyze Rh2 and F2 to form Rg3 and Rd., respectively, by forming a β-1,2-glycosidic linkage [24, 25]; UGTPg1 was characterized to glycosylate the C20-OH of PPD and PPT to produce compound K and ginsenoside F1, respectively [26]; UGTPg101 can catalyze the glycosylation of C20-OH of PPT and C6-OH of F1 to produce F1 and Rg1, respectively [27]; UGTPg100 can catalyze the glycosylation of C6-OH of PPT and F1 to form ginsenoside Rh1 and Rg1, respectively [27]. Transcription factors (TFs) play crucial roles in the regulation of secondary metabolite biosynthesis [28, 29]. Some WRKY and bHLH TFs have been found to be positive regulators of triterpene ginsenoside biosynthesis in *Panax quinquefolius* and *Panax notoginseng*, respectively [30, 31]. Until now, most of the knowledge on dammarane-type saponin biosynthesis is from *P. ginseng*, and dammarane-type saponin biosynthesis in *G. pentaphyllum* remains largely unclear.

RNA sequencing (RNA-Seq) based next-generation sequencing (NGS) technology is a powerful and economical way to discover novel genes on a large scale. This technology has been widely used to uncover genes involved in the biosynthesis of secondary metabolites. Although NGS has advantages regarding sequencing depth and cost, the short read length may restrict correct assembly and annotation [32, 33]. More recently, isoform sequencing (Iso-Seq) based on Pacific Biosciences (PacBio) SMRT technology has been developed. Compared to RNA-Seq, Iso-Seq has longer reads and an assembly-free analysis pipeline, which provide more full-length transcripts and direct evidence of the structural variation of isoforms [34, 35]. However, the higher cost and relatively low sequencing quality of Iso-Seq limit its wider application in gene mining.

Here, we provide a hybrid sequencing strategy that can produce high-quality long transcripts for gene mining and identification. Briefly, we used SMRT sequencing technology and NGS to generate comprehensive insight into full-length sequences from root, stem and leaf tissues. Moreover, we estimated the candidate enzymes involved in the biosynthesis pathway, OSCs, CYP450, and UGT through a methyl jasmonate (MeJA)-treated experiment and tissue-specific expression analyses. Finally, one OSC, one CYP450, one UGT, six WRKYs and one bHLH were selected as the candidates most likely to be involved in gypenoside biosynthesis. Our results will provide a valuable resource for investigating novel genes in the biosynthesis of gypenosides.

## Result

### Hybrid sequencing, assembly and annotation

**Fig. 2:**
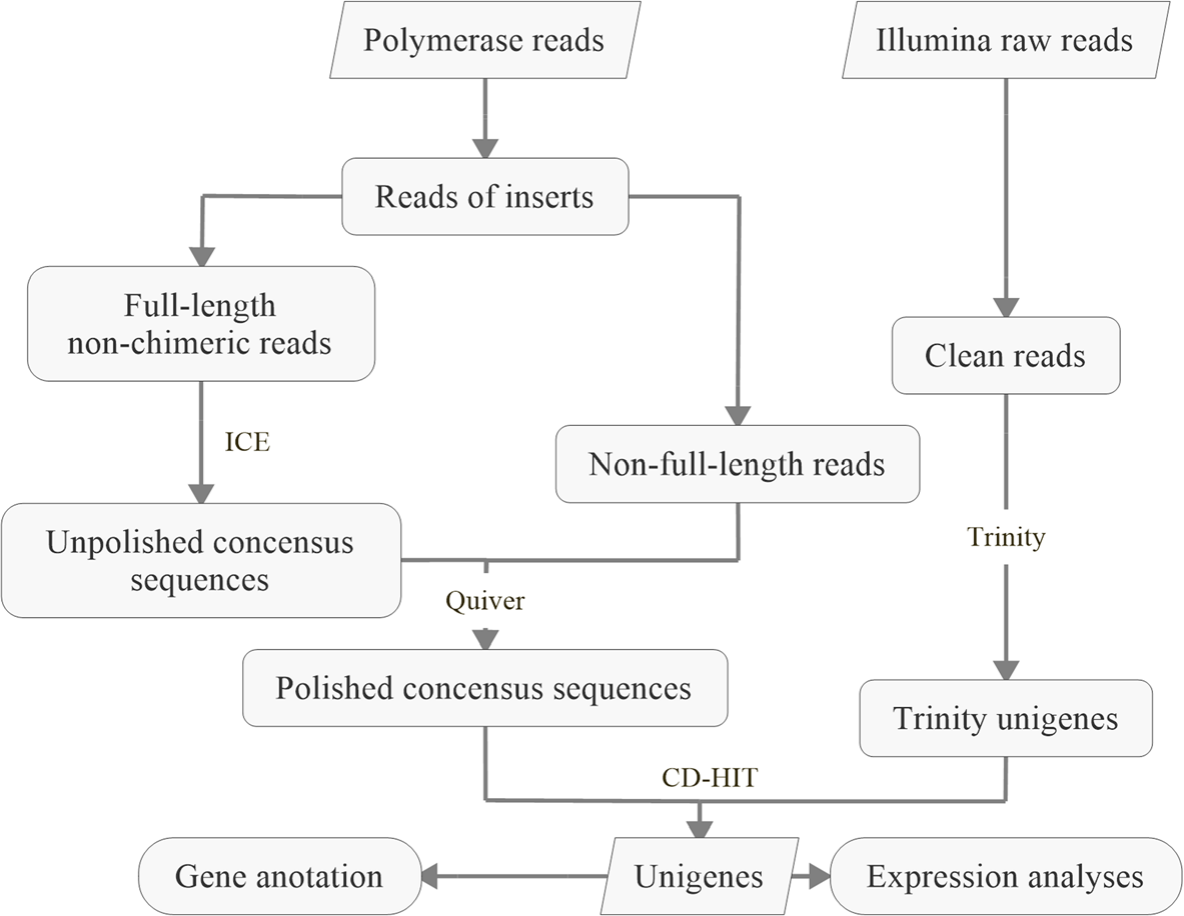
Flowchart of the experimental design and analyses for hybrid sequencing

**Fig. 3:**
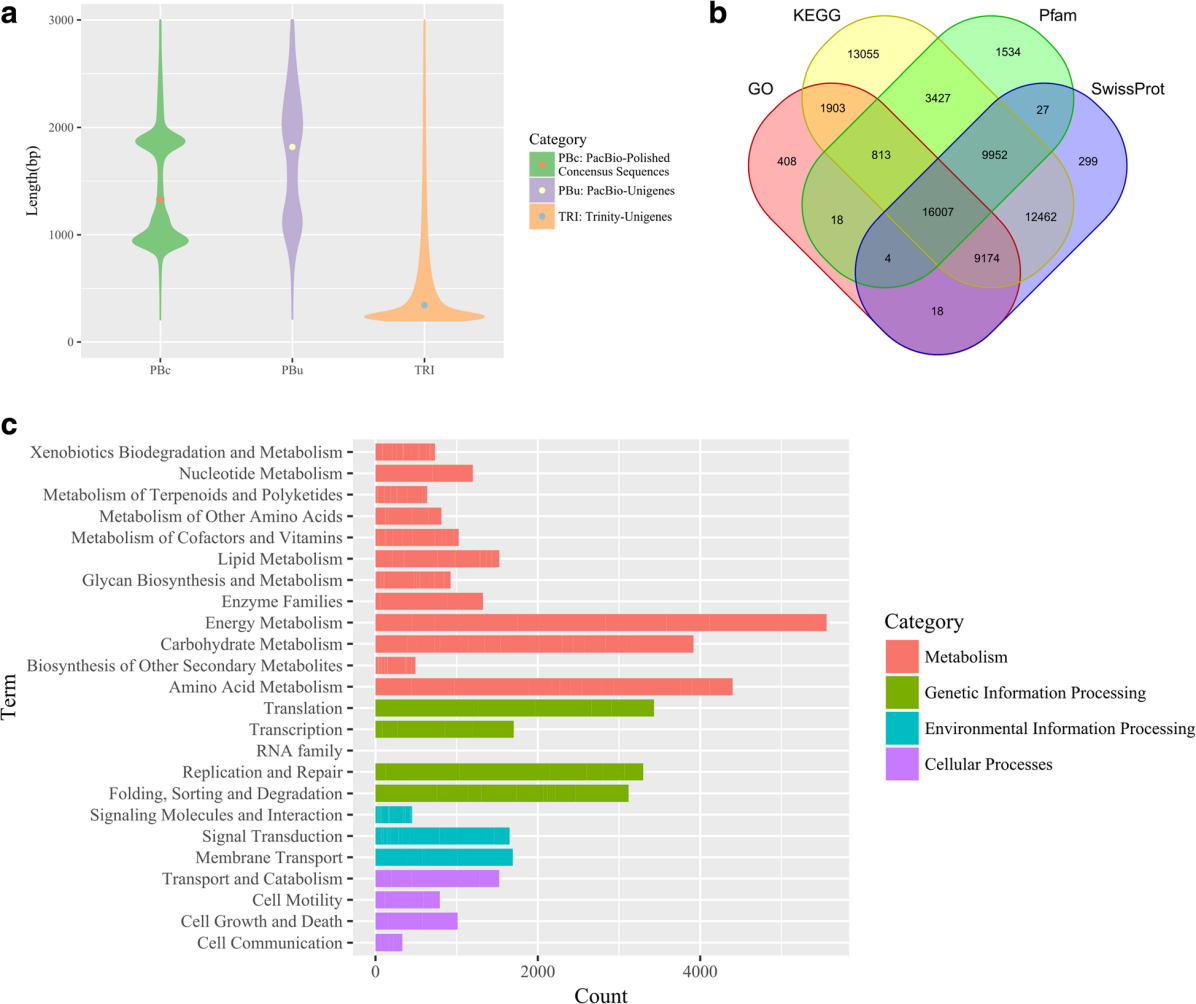
Sequence length distribution, annotation and functional classification of unigenes. **a** Sequence length distribution. **b** Venn diagram of the KEGG, SwissProt, Pfam and GO results for the *G. pentaphyllum* transcriptome. **c** Functional classification by KEGG. The abscissa indicates the number of genes annotated to the pathway, and the ordinate indicates the subcategories. The pathway is divided into four categories in this analysis, including Cellular Processes, Environmental Information Processing, Genetic Information Processing and Metabolism

To obtain as many high-quality unigenes as possible, a hybrid sequencing strategy that combined SMRT and NGS technology was used to analyze the transcriptome of *G. pentaphyllum* (Fig. 2)*.* A total of 13.17 Gb raw data were generated from eight SMRT cells by Iso-Seq. Following the PacBio standard analysis pipeline, 268,927 reads of insert were generated and then classified into 99,739 full-length non-chimeric reads and 142,079 non-full-length reads. The full-length non-chimeric sequences were clustered by ICE and then polished by Quiver to obtain 66,046 polished consensus sequences. In addition, 85.82 Gb of Illumina raw data were obtained by sequencing the transcriptomes from roots, stems, leaves and MeJA-treated leaves (Additional file 1: Table S1). To obtain more transcripts for gene discovery, all raw data from different samples were combined. After removal of adapter sequences and low-quality reads, 576,532,682 clean reads were generated. All clean reads were then assembled with Trinity software to generate 140,601 Trinity unigenes. The polished consensus sequences and Trinity unigenes were merged. Finally, after removing redundant data with CD-HIT software, 140,157 final unigenes were obtained, ranging from 200 bp to 13,760 bp in size, with an average length of 750 bp. The transcript length distribution generated using these two platforms is shown in Fig. 3a. Trinity unigenes were mostly < 500 bp, and the average length was 650 bp. PacBio polished consensus sequences and PacBio unigenes were mostly distributed in the 800–2500 bp range. Their average lengths were also longer than Trinity unigenes, at 1564 bp and 2075 bp, respectively. These results demonstrate that PacBio sequencing reads are an invaluable resource to generate longer full-length transcripts without assembly, an element of critical importance for genomic studies on species without a reference genome assembly.

All unigenes were functionally annotated by searching against the SwissProt, KEGG, Pfam and GO databases. The results indicated that 69,101 (49.3%) unigenes showed annotation hits in at least one of four databases, and 16,007 unigenes showed annotation hits in all databases (Fig. 3b). In total, 126,493 unigenes were assigned GO terms, which were classified into three major categories (molecular function, cellular component and biological process; Additional file 2: Figure S1). In the biological process category, the major subgroups were “metabolic process” (GO: 0008152) and “cellular process” (GO: 0009987). In the cellular component category, unigenes categorized in the “cell” (9416, 7.44% of the total), “membrane” (9354, 7.39% of the total) and “cell part” (9258, 7.31%) were highly represented. In the molecular function category, the major categories were “catalytic activity” (GO:0003824) and “binding” (GO: 0005488). Furthermore, based on KEGG annotation, 41,549 unigenes were categorized into four major categories with different functions (Cellular Processes, Environmental Information Processing, Genetic Information Processing and Metabolism). Unigenes related to energy metabolism represented the largest subcategory, followed by genes related to amino acid metabolism and carbohydrate metabolism (Fig. 3c). More importantly, 614 unigenes were annotated as related to terpenoid metabolism and polyketides, accounting for 2.7% of the metabolism category, and 190 unigenes were related to terpenoid backbone biosynthesis. These findings are helpful for mining the candidate genes involved in gypenoside biosynthesis.

### Candidate OSCs involved in gypenoside biosynthesis

OSCs catalyze the first committed step in triterpene biosynthesis, namely, the cyclization of the universal triterpene precursor 2,3-oxidosqualene and therefore define sterol and triterpene skeletal diversity. More than 100 diverse triterpene skeletons are currently known in plants. In this study, we obtained 5 full-length candidate OSCs after data mining and manual curation (GpOSC1–5). Alignment analysis showed that all candidates contained six repeats of QW (QXXXGXW) motifs and one DCTAE motif (Additional file 3: Figure S2). The former can enhance the structure of the protein and stabilize the carbocation intermediates during cyclization, while the latter is presumed to be responsible for the initiation of the cyclization reaction [36, 37]. Additionally, GpOSC2 and GpOSC4 have one MWCHCR motif, within which the histidine residue has been presumed to be important for stabilization of the protosteryl cation intermediate [38]. The protosteryl cation intermediate is catalyzed by the enzymes cucurbitadienol synthase (CbQ), CAS and LS.

**Fig. 4:**
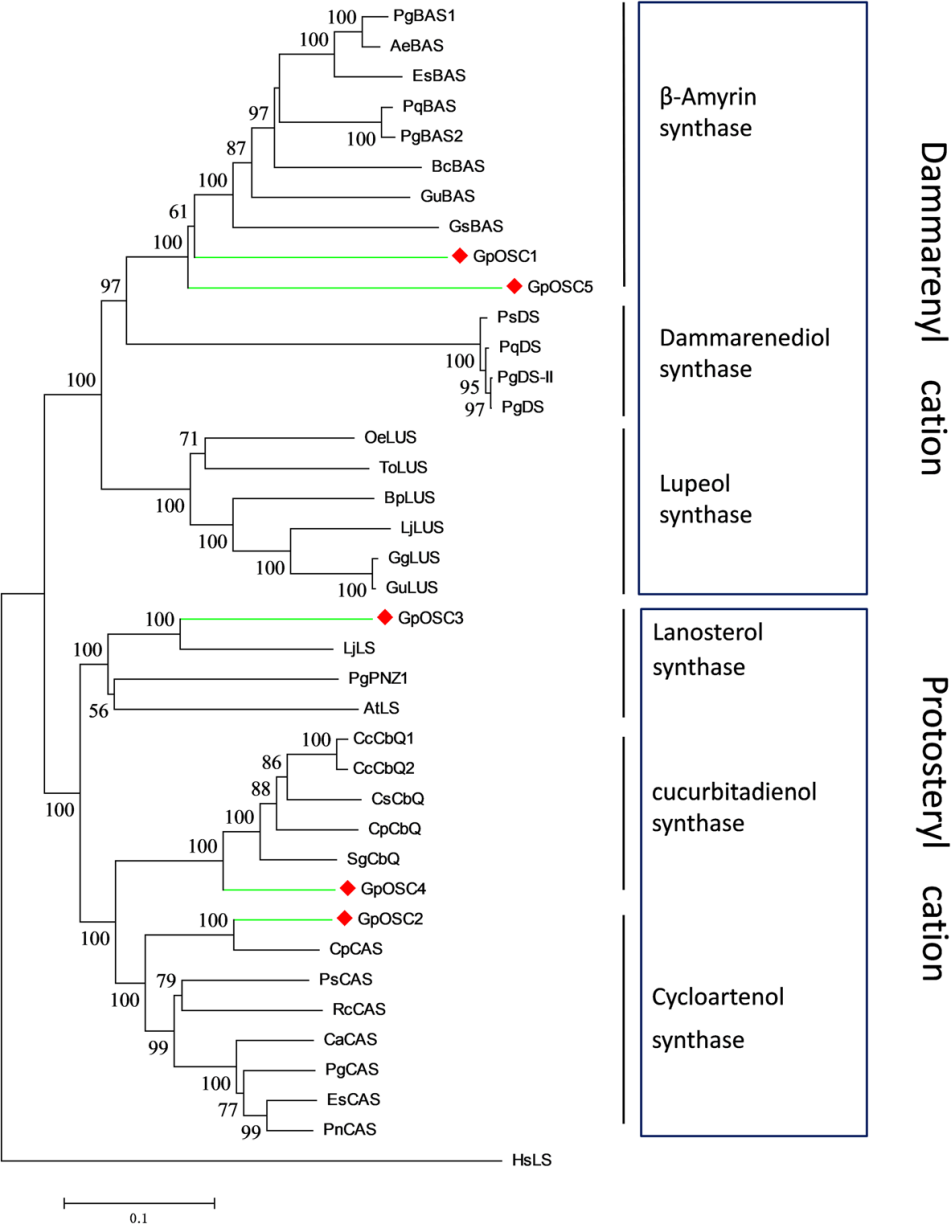
Phylogenetic analyses of the GpOSCs from *G. pentaphyllum* and characterized OSCs from other plants. Amino acid sequences were aligned using the program ClustalW, and evolutionary distances were computed using the Poisson correction method with MEGA6. Lanosterol synthase from *Homo sapiens* (HsLS) is used as the outgroup. Values less than 50% are not shown. The GenBank accession numbers of the sequences are EsCAS (AFC67276), PnCAS (ABY60426), CaCAS (AAS01524), PgCAS (BAA33460), RcCAS (Q2XPU6), PsCAS (BAA23533), CpCAS (Q6BE25), AtLS (BAE95408), LjLS (BAE95410), PgPNZ1 (BAA33462), HsLS (AAB36220), BpLUS (BAB83087), LjLUS (BAE53430), ToLUS (BAA86932), GgLUS (BAD08587), GuLUS (BAL41371), OeLUS (BAA86930), PqDS (AGI15962), PgDS (AEO27862), PsDS (ANB82450), PgDSII (BAF33291), AeBAS (ADK12003), EsBAS (APZ88354), BcBAS (ADM89633), PgBAS1 (O82140), GsBAS (ACO24697.1), and PqBAS (AGG09939)

To further predict the function of these OSCs, we analyzed the phylogenetic relationship between the *G. pentaphyllum* OSCs and characterized OSCs in other plants (Fig. 4). In the phylogenetic tree, LS, CAS and CbQ group together, while DS, BAS and lupeol synthase (LUS) form another group, which is in concordance with previous studies. The two groups of OSCs have different catalytic intermediates. LS, CAS and CbQ can direct 2,3-oxidosqualene to the chair-boat-chair (CBC) conformation and then form the protosteryl cation when initiating the cyclization reaction. In contrast, DS, BAS and LUS can direct this substrate to the chair-chair-chair conformation (CCC) and then form dammarenyl cations. In general, sterol skeletons are synthesized through the CBC cyclization pathway, and triterpene skeletons are synthesized through the CCC cyclization pathway. As shown in Fig. 4, GpOSC2 belonged to the CAS clade, which showed 88% identity with the CAS from *Cucurbita pepo*, demonstrating that GpOSC2 might have a similar catalytic activity with CAS. GpOSC3 belonged to the LS clade and that it had 77% identity with the LS from *Luffa aegyptiaca*. GpOSC4 is predicted as a CbQ, which can catalyze 2,3-oxidosqualene to cyclize to cucurbitacins. Cucurbitacins are widely distributed in plants in the Cucurbitaceae family. GpOSC1 and GpOSC5 phylogenetically belonged to the BAS clade and had 70 and 64% identity with the identified BAS of *P. quinquefolius*, respectively*.* Simultaneously, GpOSC1 had 57% identity with DS from *P. ginseng.* However, no β-amyrin-type saponins were isolated from *G. pentaphyllum,* and the two enzymes need further functional characterization.

**Fig. 5:**
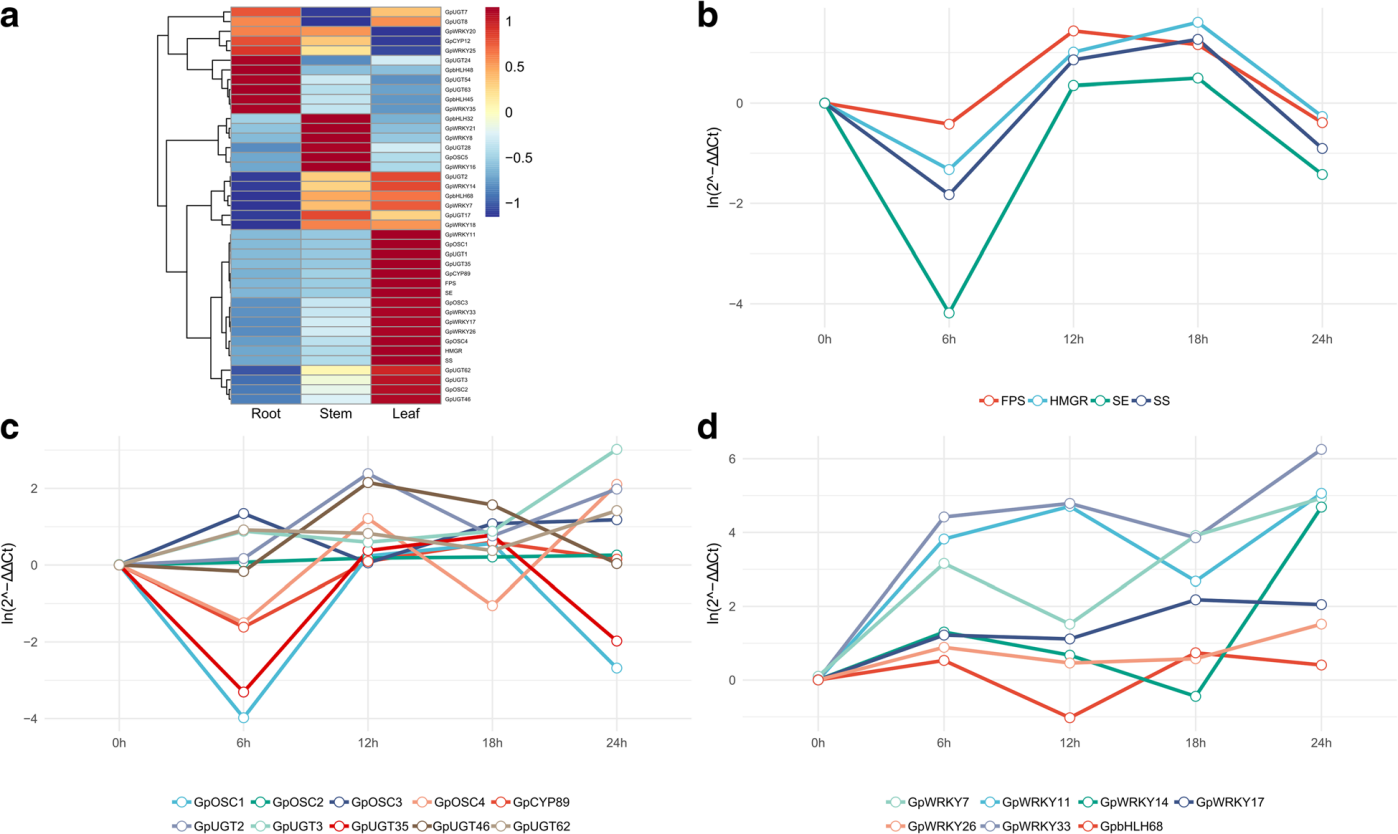
Expression analysis of the selected *GpOSCs*, *GpCYP450s*, *GpUGTs*, *GpWRKYs* and *GpbHLHs* in different tissues and MeJA-treated leaves by real-time PCR. **a** Heatmap of expression levels based on their real-time PCR analysis in three tissues, including roots, stems, and leaves. **b** Expression analysis of the upstream genes in MeJA-treated leaves by real-time PCR. Here, five time points, 0 h, 6 h, 12 h, 18 h and 24 h, and 0 h were used as a reference. **c** Expression analysis of the *GpOSCs*, *GpCYP450* and *GpUGTs* in MeJA-treated leaves by real-time PCR. **d** Expression analysis of the *GpWRKYs* and *GpbHLH* in MeJA-treated leaves by real-time PCR

In general, the genes encoding enzymes in the same pathway are coexpressed. Therefore, with the four known genes involved in triterpene biosynthesis as references, we analyzed the expression levels of the 5 *GpOSCs* in different tissues using real-time PCR (Fig. 5a). 3-hydroxy-3-methyl glutaryl coenzyme A reductase (*HMGR*), *FPS*, *SE* and *SS* were found to be tightly coexpressed; they were all expressed highest in leaves, followed by stems and then roots. The results agree with the gypenoside contents in these tissues. *GpOSC5* was expressed highest in stems*;* other *GpOSCs* had similar expression patterns to those of the four upstream genes, although *GpOSC3* had much lower expression levels than *GpOSC1*, *GpOSC2* and *GpOSC4*. In particular, the expression of *GpOSC1* in leaves was 551.84-fold greater than that in roots and 33.38-fold greater than that in stems.

In plants, MeJA is an important signaling molecule in response to abiotic or biotic stresses, which can induce the biosynthesis of many secondary metabolites for plant defense [39]. MeJA can induce the expression of genes involved in the ginsenoside synthetic pathway, such as *FPS*, *SS*, *SE* and *DS*, thereby increasing the content of ginsenosides in *P. ginseng* [40, 41]. This study showed that *HMGR*, *FPS, SE* and *SS* in the gypenoside biosynthetic pathway can also be induced by MeJA in *G. pentaphyllum* (Fig. 5b). More interestingly, the expression of all the genes was depressed at 6 h after MeJA treatment and then increased gradually. Among them, *SS*, *SE* and *HMGR* increased between 6 h and 18 h, reaching a maximum expression of 3.54-fold, 1.64-fold and 4.98-fold greater than that at 0 h, respectively. *FPS* sharply increased between 6 and 12 h, peaking at 12 h with a 4.20-fold increase. Among the four *GpOSCs,* only *GpOSC2* was not induced by MeJA. *GpOSC1* had similar expression patterns with the four known genes in the gypenoside biosynthetic pathway. In particular, *GpOSC1* and *HMGR* showed the highest similarity in expression pattern with the Spearman correlation coefficient of 1, suggesting their consistency in response to MeJA induction (Fig. 5c). According to coexpression and phylogenetic analyses, we speculated that GpOSC1 most likely functions as a DS.

### Candidate CYP450s involved in gypenoside biosynthesis

CYP450s that catalyze structural modification are crucial for the diversification and functionalization of triterpenes. Based on the annotation results, 145 CYP450s with lengths from 300 to 581 amino acids were identified in *G. pentaphyllum* (GpCYP1–145). They were classified by alignment with the CYP450 database using standard sequence similarity cutoffs, specifically 40, 55 and 97% for family, subfamily and allelic variants, respectively [42]. Thus, the 145 GpCYP450s were classified into 40 families and 39 subfamilies (Additional file 4: Figure S3). However, seven CYP450s (GpCYP3, 50, 51, 83, 96, 97, 114) did not belong to any old family. We considered that they belonged to novel P450 families. In addition, 68 CYP450s were found to belong to novel P450 subfamilies.

**Fig. 6:**
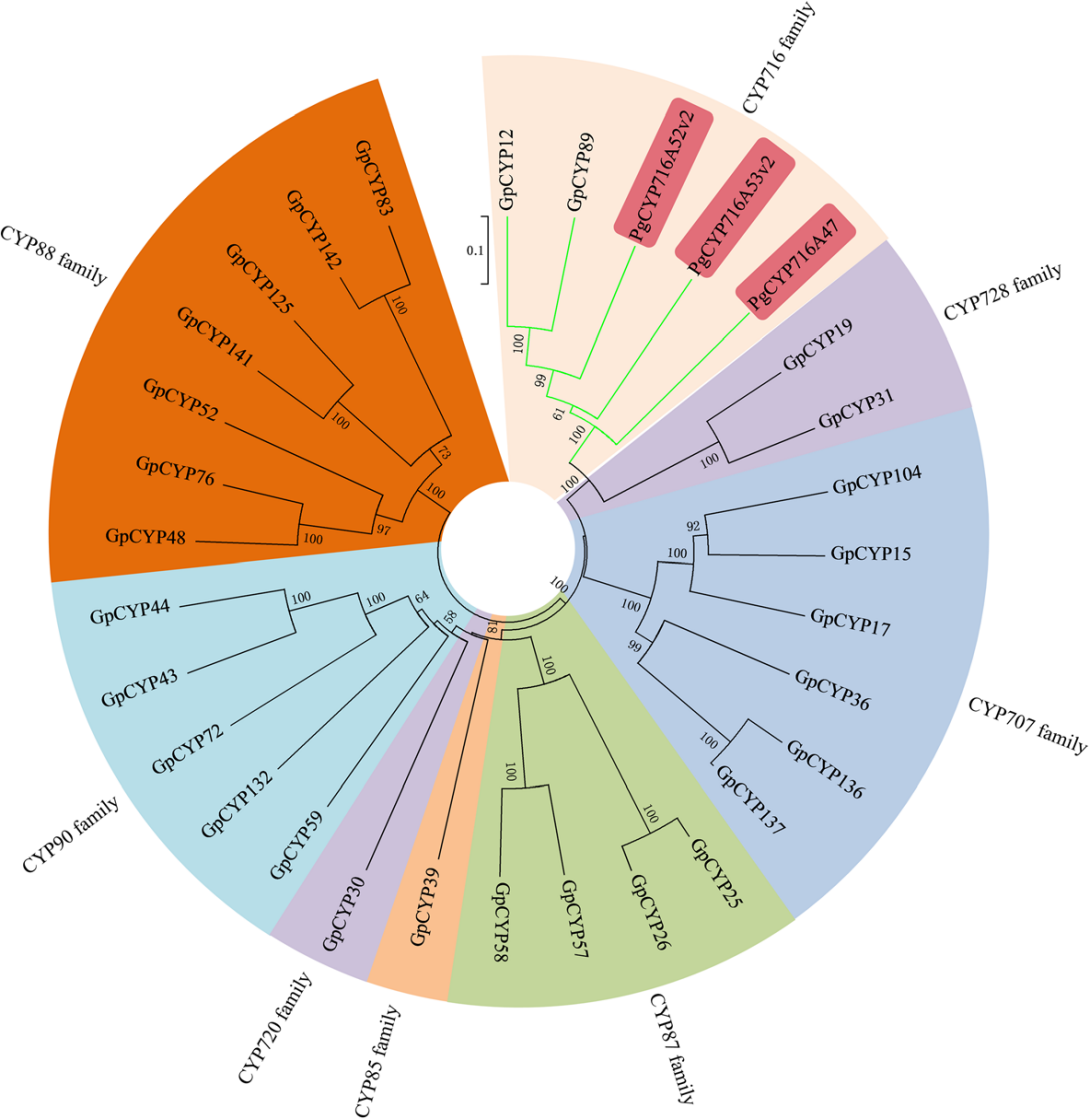
Phylogenetic analyses of the GpCYP450s belonged to 85 clans from *G. pentaphyllum* and characterized CYP450s from *P. ginseng*. The GenBank accession numbers of the sequences are PgCYP716A47 (AEY75212.1), PgCYP716A53v2 (AFO63031.1), and PgCYP716A52v2 (AFO63032.1)

To date, all characterized CYP450s involved in ginsenoside biosynthesis belonged to the CYP716A subfamily (CYP716 family, 85 Clan). We found that two GpCYP450s (GpCYP12 and GpCYP89) from *G. pentaphyllum* belong to the CYP716 family. Therefore, phylogenetic analysis was carried out with the 85 Clan GpCYP450s and three characterized CYP450s involved in ginsenoside biosynthesis from *P. ginseng* (Fig. 6). Considering that almost all gypenosides are protopanaxadiol-type saponins, GpCYP450s with high identity with protopanaxadiol synthase (CYP716A47) are most likely involved in gypenoside biosynthesis. GpCYP12 and GpCYP89 had 49 and 48% identity to CYP716A47, respectively. However, they had higher identity (approximately 57%) with β-amyrin-28 oxidase (CYP716A52v2) of *P. ginseng,* although we have not found any β-amyrin-type triterpene saponins from *G. pentaphyllum* until now.

The expression patterns of these two *GpCYP450* genes in different tissues and MeJA-treated leaves were then further analyzed with real-time PCR. As shown in Fig. 5a, *GpCYP89* had a similar tissue-specific expression pattern to that of the upstream genes. The expression of *GpCYP89* in leaves was more than 11.53-fold higher than that of stems, and 265.41-fold higher than that of roots. Unsurprisingly, *GpCYP89* can be induced by MeJA because most *CYP450s* are involved in secondary metabolism in plants (Fig. 5c). *GpCYP89* had similar expression patterns to those of *SS*, *SE* and *HMGR*, which were downregulated before 6 h and then continuously increased and reached the highest expression at 18 h; this highest expression was 1.82-fold greater than that at 0 h, with Spearman correlation coefficients of 0.9 with *HMGR*. According to phylogenetic and expression analyses, GpCYP89 from the CYP716 family may have functioned as protopanaxadiol synthase in *G. pentaphyllum*.

### Candidate UGTs involved in gypenoside biosynthesis

**Fig. 7:**
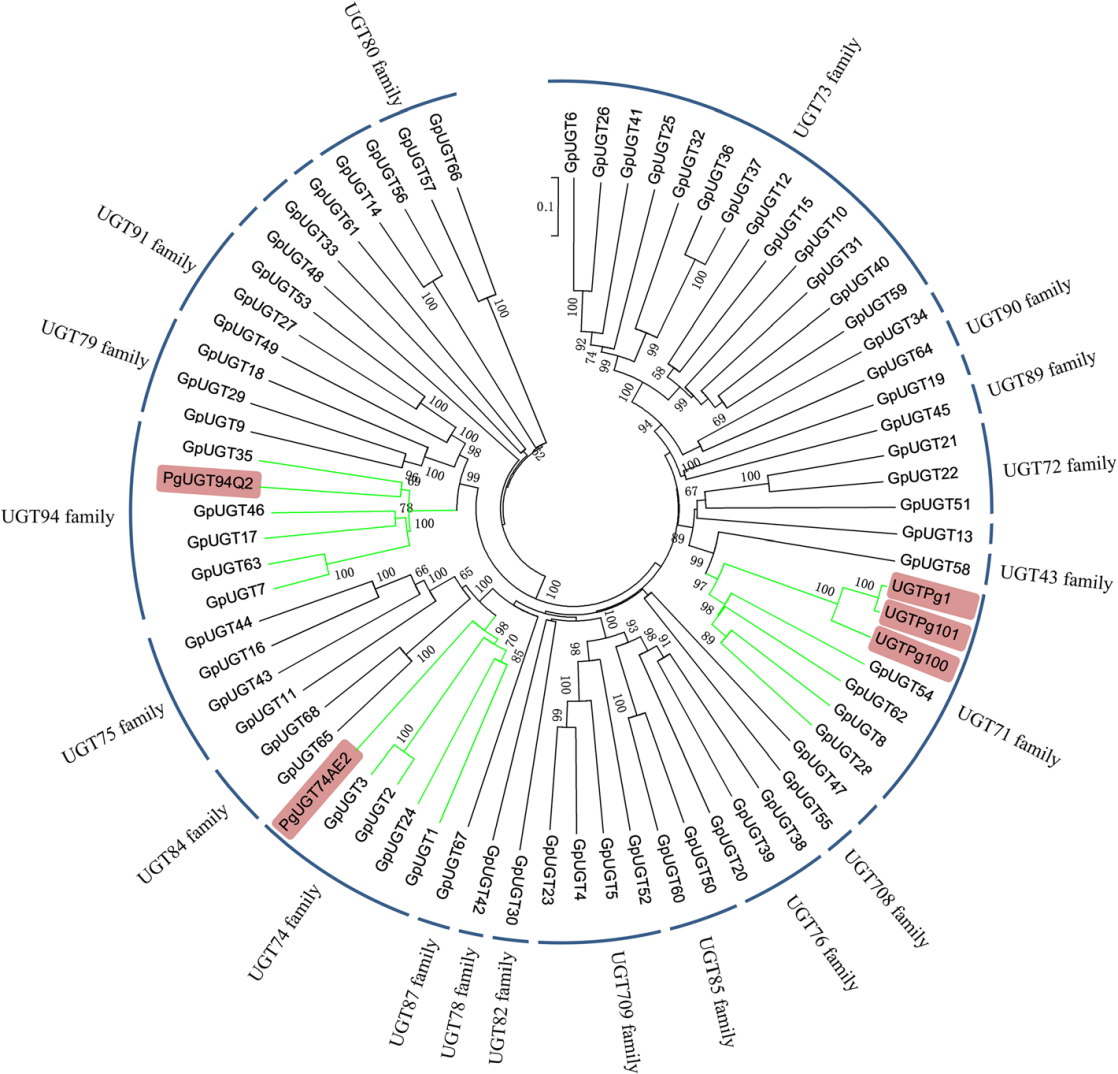
Phylogenetic analyses of 68 GpUGTs from *G. pentaphyllum* and characterized UGTs from *P. ginseng.* The GenBank accession numbers of the sequences are PgUGT74AE2 (AGR44631.1), PgUGT94Q2 (AGR44632.1), UGTPg1 (AIE12479.1), UGTPg100 (AKQ76388.1), and UGTPg101(AKQ76389.1). Those who did not indicate the family belonged to the novel family

Glycosylation is the last step in gypenoside biosynthesis. In this study, we obtained a total of 254 unigenes annotated as UGTs. We selected 68 UGTs with peptide lengths greater than 352 amino acids (GpUGT1–68) and divided them into 20 UGT families based on the criteria of amino acid sequence identity cutoffs, briefly, 40% for family, and 60% for subfamily [43]. Nine GpUGTs were noted as novel UGT families (Fig. 7).

To date, five UGTs from *P. ginseng* have been shown to be involved in ginsenoside biosynthesis, which belong to the UGT71, UGT74 and UGT94 families. Phylogenetic analysis showed that a total of 13 GpUGTs clustered into the above three families (Fig. 7). Therefore, they are candidate UGTs for gypenoside biosynthesis. Among them, GpUGT35 was clustered into the UGT94 family with the highest identity (50%) to PgUGT94Q2. GpUGT24 was clustered into the same branch as PgUGT74AE2 with 42% identity. GpUGT8 showed the highest identity (46%) with UGTPg1 (UGT71 family) from *P. ginseng*.

According to phylogenetic analysis, 13 *GpUGTs* were chosen for analysis of their expression patterns by real-time PCR. In the tissue-specific pattern assay, five *GpUGTs* (*GpUGT2*, *GpUGT3*, *GpUGT35*, *GpUGT46* and *GpUGT62*) showed strong similarity to *FPS*, *SS*, *SE* and *HMGR* (Fig. 5a). In particular, the expression of *GpUGT35* in leaves was 28.00-fold higher than that in stems and 453.00-fold higher than that in roots. In the MeJA treatment experiment, the expression of all five *GpUGTs* can be regulated by MeJA (Fig. 5c). However, only *GpUGT35* had a similar expression pattern to *SS*, *SE* and *HMGR*, which was continuously increased by MeJA between 6 h–18 h and then reached the highest at 18 h, which was 2.17-fold compared to that at 0 h. The Spearman correlation coefficient was 1 with *HMGR*. According to the phylogenetic and expression analysis, GpUGT35 from the UGT94 family was regarded as a candidate enzyme responsible for gypenoside biosynthesis and will be the subject of further study.

### Candidate TFs possibly regulating gypenoside biosynthesis

**Fig. 8:**
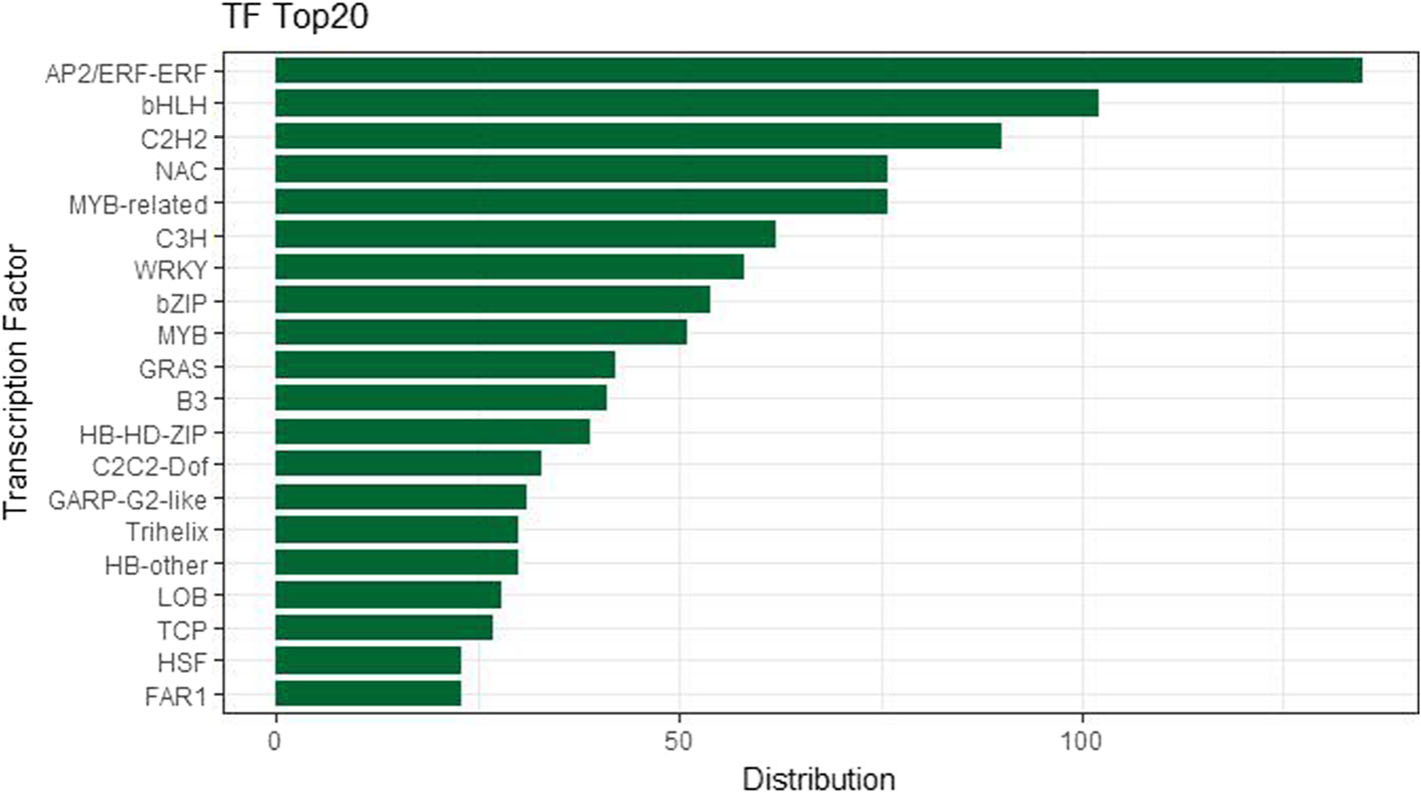
Distribution of transcription factors

In this study, we selected 1362 TFs distributed in 64 families, including ARF, B3, MYB, bHLH, bZIP, and WRKY. As shown in Fig. 8, the largest family is AP2/ERF-ERF, accounting for 9.9% of the total, followed by bHLH and C2H2, accounting for 7.5 and 6.6%, respectively. To date, only two TFs have been found to be involved in the regulation of the triterpenoid pathway, PqWRKY1 from *P. quinquefolius* and PnbHLH from *P. notoginseng* [30, 31].

WRKY proteins are one of the largest families of TFs in plants. The WRKY domain can specifically bind to the target gene promoter W-box sequence to regulate the expression of target genes [44]. PqWRKY1 is reported as a positive regulator related to gypenoside biosynthesis in American ginseng [30]. A total of 58 putative WRKY proteins were identified by transcriptome-wide identification from *G. pentaphyllum*, among which 38 WRKYs (GpWRKY1–38) had complete WRKY domains (Additional file 5: Figure S4). According to Eulgem’s method, 38 GpWRKYs were categorized into Groups I, II and III, and Group II can be further classified into subgroups IIa, IIb, IIc, IId and IIe. (Additional file 6: Table S2) [45]. PqWRKY1 belonged to IIc, and 13 GpWRKYs from *G. pentaphyllum* were clustered into this subgroup. The expression of 13 *GpWRKY*s was analyzed with real-time PCR. Among them, six *GpWRKYs* (*GpWRKY7*, *GpWRKY11*, *GpWRKY14*, *GpWRKY17*, *GpWRKY26* and *GpWRKY33*) showed the highest expression in leaves, compared with roots and stems (Fig. 5a). Furthermore, the expression of all of these 6 *GpWRKYs* was increased by MeJA treatment and reached the highest level (> 4.56-fold) at 24 h compared with that at 0 h (Fig. 5d).

bHLH proteins are one of the largest families of TFs in both plants and animals, and play important roles in plant metabolism [46]. PnbHLH1 from *P. notoginseng* is regarded as another positive regulator involved in ginsenoside biosynthesis [31]. A total of 89 TFs with complete bHLH domains (Additional file 7: Figure S5) were grouped into 21 subgroups by phylogenetic tree construction [47, 48], with the XII subgroup having the largest number of members (13 sequences) and the VIIIa, II, and IIIf subgroups having the fewest (1 sequence) (GpbHLH1–89; Additional file 8: Table S3). PnbHLH1 belonged to IVa and four GpbHLHs (GpbHLH32, GpbHLH45, GpbHLH48, GpbHLH68) clustered with PnbHLH1 together. The expression of four GpbHLHs was then analyzed with real-time PCR. As shown in Fig. 5a, only *GpbHLH68* was highly expressed in leaves. Moreover, *GpbHLH68* expression was increased by MeJA treatment and reached the highest level (~ 2-fold) at 18 h compared with that at 0 h, suggesting its possible positive role in regulating gypenoside biosynthesis (Fig. 5d). Based on the above phylogenetic and expression analysis, six GpWRKYs and one GpbHLH were regarded as possible positive regulators of gypenoside biosynthesis and will be the subject of further study.

## Discussion

Transcriptome sequencing is a powerful and economical tool for obtaining genetic information on a large scale in organisms without available genome sequences. Undoubtedly, low-cost NGS has provided deep insights into the characterization and quantification of the transcriptome; however, the relatively short reads generated by NGS have prohibited the accuracy of transcript reconstruction after assembly with software. In most cases, this assembly step may suffer from a misassembly of the sequences, lowering the accuracy of gene annotation and expression quantification in the following steps. The third generation sequencing technology, SMRT sequencing developed by PacBio, offers an alternative way to overcome this limitation. SMRT sequencing with a read length up to 20 kb renders the PacBio platform very effective in the sequencing of full-length cDNAs that exhibit long-transcript isoforms, avoiding the mistakes introduced by assembly steps [49]. However, the sequencing accuracy of SMRT technology is much lower than that of NGS. An alternative way to overcome these limitations is to integrate NGS short reads and PacBio long reads via hybrid sequencing. In hybrid sequencing, NGS short reads are mapped to and correct SMRT reads and could improve the accuracy of gene isoform identification and abundance estimation.

Natural products are important sources for drug discovery. Modern pharmacological studies have shown that ginsenosides have great potential in the prevention and treatment of multiple cancers [3]. Ginsenosides are generally produced by plants from the *Panax* genus, such as *P. ginseng*, *P. quinquefolius* and *P. notoginseng*. However, these plants grow slowly and cannot be continuously cropped in the same fields [12]. Thus, the ginsenosides isolated from the *Panax* plants are not able to meet the need for drug production and development. *G. pentaphyllum* can produce some triterpenoids named gypenosides, which have structures that are the same as or similar to those of ginsenosides [11]. Compared to *Panax* plants, *G. pentaphyllum* grows much faster, can be harvested four times a year and has a higher triterpenoid content. Therefore, this plant is a promising alternative resource for ginsenoside production. In addition, because of its short life cycle, easy tissue culture and genetic transformation, it is also an emerging medicinal model plant for triterpenoid biosynthesis.

Most ginsenosides and gypenosides have dammarane-type aglycones, which are not widely distributed triterpenoids, mainly produced by the *Panax* and *Gynostemma* genera [4–7]. Interestingly, even though the two genera belong to different evolutionary branches that are very far from each other, they can produce the same triterpenoids. DS is the signature enzyme of the dammarane biosynthetic pathway [16]. We found five OSCs in the *G. pentaphyllum* transcriptomes. Phylogenetic analysis showed that no GpOSCs clustered with DSs from the *Panax* genus, while two GpOSCs belonged to the BAS group, the group nearest to the DS group in the phylogenetic tree, among which GpOSC1 showed the highest similarity with the characterized DS (Fig. 4). Since the genes in the same biosynthetic pathway are generally coexpressed, we compared the expression patterns of all *GpOSCs* with four upstream genes encoding *HMGR*, *FPS*, *SE* and *SS* and found that the expression patterns of GpOSC1 were the most similar to those of the upstream genes (Fig. 5). Combined phylogenetic and expression analyses were used to screen two tailoring enzymes, GpCYP89 and GpUGT35, which may be involved in gypenoside biosynthesis. GpCYP89 belongs to the CYP716 family. To date, approximately ten enzymes from the CYP716 family have been reported to be involved in triterpenoid oxidation reactions, such as CYP716A86 and CYP716A83 from *Centella asiatica*, CYP716A111 from *Aquilegia coerulea* and CYP716A141 from *Platycodon grandiflorus*, suggesting that CYP716 enzymes may play an important role in triterpenoid biosynthesis in eudicots [50]. GpUGT35 belongs to the UGT94 family. It was reported that, in this family, SgUGT94–289-2 from *Siraitia grosvenorii* can glycosylate mogroside M2-E and mogroside M3 at the C-24 position to yield mogroside M3x and mogroside Sia [51, 52]. Further study is needed to elucidate the roles of GpCYP89 and GpUGT35 in gypenoside biosynthesis.

TFs play an important role in response to biotic and abiotic stress, and may further regulate the biosynthesis of secondary metabolites [53]. In our study, we annotated 1362 TFs and further identified six GpWRKYs and one GpbHLH as candidates for the regulation of gypenoside biosynthesis. The six GpWRKYs all come from subgroup IIc. In this subgroup, it was reported that some WRKYs can regulate secondary metabolite biosynthesis. For example, CjWRKY1 in *Coptis japonica* is a positive regulator of berberine biosynthesis through increasing the level of transcripts of pathway genes [54]. The GpbHLH candidate belongs to subgroup IVa. In this subgroup, CrBIS1 in *Catharanthus roseus* is reported to increase the content of iridoids and MIAs in *C. roseus* by transactivating the expression of genes encoding the enzymes that catalyze geranyl diphosphate to iridoid loganic acid [55]. Further analysis is needed to verify the predicted roles of these WRKYs and bHLH in the regulation of gypenoside biosynthesis.

## Conclusions

*G. pentaphyllum* is not only a promising gypenoside or ginsenoside provider but also an emerging medicinal model plant for triterpenoid biosynthesis. Here, we first combined PacBio sequencing and Illumina sequencing (hybrid sequencing) to mine the uncharacterized genes encoding enzymes involved in gypenoside biosynthesis in *G. pentaphyllum*. Highly accurate full-length transcripts produced by hybrid sequencing can significantly improve the accuracy of gene annotation and gene expression quantification, which will be helpful for gene discovery related to gypenoside biosynthesis. Hybrid sequencing first provided abundant genetic information resources for *G. pentaphyllum*, laying an important foundation for research on genetic breeding, metabolic regulation, synthetic biology and other aspects of *G. pentaphyllum*. In addition, combined phylogenetic and coexpression analyses identified one OSC, one CYP450, one UGT, six WRKYs and one bHLH from the *G. pentaphyllum* transcriptomes to be the lead candidates involved in gypenoside biosynthesis. These results will provide new insights into gypenoside biosynthesis.

## Methods

### Plant materials and sample preparation

Plant materials of *G. pentaphyllum* (two years old) were grown in the Institute of Medicinal Plant Development (Additional file 9: Figure S6). Roots, stems, leaves, and MeJA-treated leaves of *G. pentaphyllum* were collected. The induced leaves were sprayed with 200 μm MeJA and collected at different treatment times (0 h, 6 h, 12 h, 18 h and 24 h). After collection, all samples were immediately frozen in liquid nitrogen and stored at − 80 °C until RNA was extracted.

### RNA extraction

Total RNA was extracted using the RNAprep Pure Kit for plant (Tiangen Biotech, China) and quantified by Qubit (Invitrogen^TM^ Life Technologies, USA). The RNA integrity was evaluated with an Agilent 2100 Bioanalyzer (Agilent Technologies, USA).

### PacBio sequencing

The total RNA from different tissues was equally mixed together. PolyA RNAs were isolated from total RNA using Dynal oligo (dT) 25 beads (Life Technologies, USA) and subjected to Iso-Seq library construction. Briefly, cDNA was synthesized from the polyA RNA using the SMARTer^®^ PCR cDNA Synthesis Kit (Clontech, Mountain View, CA, USA). Size selection was carried out on a BluePippin (Sage Science, USA), and 0.8–2 kb, 2–3 kb, 3–6 kb fractions were collected. For each fraction, a SMRTbell template library was prepared and sequenced using the PacBio RSII platform. A total of eight SMRT cells were carried out in this study.

### Illumina sequencing

Illumina sequencing libraries were constructed with the root, stem and leaves from *G. pentaphyllum* with three replicates. In addition, MeJA-treated leaves at different times (0 h, 6 h, 12 h, 24 h) were collected to construct Illumina sequencing libraries. These 13 transcriptome libraries were paired-end sequenced using the Illumina NextSeq 500 system.

### Bioinformatics pipeline for unigene generation

The high-quality reads of insert (circular consensus sequences) were generated from SMRT raw data and classified into full-length non-chimeric reads and non-full-length reads with SMRT Analysis software v2.3.0 [56]. ICE and Quiver were used to cluster and polish the full-length non-chimeric sequences to generate the polished consensus sequences. Illumina clean reads were obtained after the adapters, and low-quality sequences were filtered out from the raw data. These clean data were assembled into Trinity unigenes with the Trinity assembler [57]. Finally, the polished consensus sequences and Trinity assembled unigenes were merged together, and redundant data were removed with CD-HIT software (-T 12 -M 45000 -c 0.85) to obtain final unigenes.

### Functional annotation

BLAST was applied to search against The Kyoto Encyclopedia of Genes and Genomes (KEGG, https://www.genome.jp/kegg) [58] and SwissProt (http://www.UniProt.org) databases with E-value <1e-5 [59]. Hmmscan was performed to search against the Pfam database (https://pfam.xfam.org) with E-value < 0.01 [60]. Gene Ontology (GO, http://geneontology.org) terms were assigned to each unigene using Blast2Go based on the best BLASTx hit from the NR database [61]. The iTAK program (version 1.7.0b) (http://itak.feilab.net) was used for identification and classification of TFs [62]. The Venn diagram was plotted with the VennDiagram package in R [63].

### Phylogenetic analysis

Amino acid sequences were aligned using the ClustalW program, and evolutionary distances were computed using the pairwise deletion method, and a neighbor-joining (NJ) tree was constructed with MEGA6 [64]. Bootstrap values obtained after 1000 replications are given on the branches. Values less than 50% are not shown.

### Qualitative analysis by real-time PCR analysis and heatmap visualization

RNA samples were isolated from roots, stems, leaves and MeJA-treated leaves (0 h, 6 h, 12 h, 18 h and 24 h). Reverse transcription was performed using the GoScript™ Reverse Transcription System Kit (Promega, USA). Primers were designed using Primer3 version 4.1.0 (http://primer3.ut.ee). The primers used in this study are listed in Additional file 10: Table S4. RT-PCR analyses were then conducted using a Bio-Rad CFX96 RT-PCR system. The reaction mixture (20 μL) contained 10 μL 2 × SYBR Premix Ex Taq (Takara, Tokyo, Japan), 0.5 μL each of the forward and reverse primers and 1 μL of template cDNA. PCR amplification was performed under the following conditions: 95 °C for 30 s; 40 cycles of 95 °C for 5 s, 60 °C for 30 s and 72 °C for 15 s; 95 °C for 10 s. The heatmap was plotted with the pheatmap package in R [65]. Here, the upstream gene SE was used as a reference and the 2^-∆∆Ct^ values were plotted with scale = “row” enabled.

### WRKY and bHLH domain analysis

The domains of WRKY and bHLH were analyzed by MEME (http://meme-suite.org). Then, the alignment of the conserved parts was trimmed out and submitted to Skylign (http://skylign.org) to generate their corresponding sequence logos.

## Additional files

Additional file 1: **Table S1.** The libraries for PacBio and Illumina sequencing. A total of 13 sequencing libraries. PacBio sequencing includes three libraries. The fragment length was 0.8–2 kb, 2–3 kb, and 3–6 kb. Illumina sequencing libraries were constructed with the root, stem and leaves from *G. pentaphyllum* with three replicates. MeJA-treated leaves at different times (0 h, 6 h, 12 h, 24 h) were collected to construct Illumina sequencing libraries. (XLSX 9 kb)

Additional file 2: **Figure S1.** GO annotation of unigenes. (PNG 200 kb)

Additional file 3: **Figure S2.** Multiple sequence alignments of candidate OSCs. Candidate OSCs were subjected to multiple sequence alignments. The red solid line indicates the QW motif, the rounded rectangle indicates the MWCHCR motif and the red rectangle indicates the DCTAE motif. (PNG 1760 kb)

Additional file 4: **Figure S3.** Phylogenetic analyses of the 145 GpCYP450s from *G. pentaphyllum* and characterized CYP450s from *P. ginseng*. (PNG 928 kb)

Additional file 5: **Figure S4.** Sequence logo of GpWRKY domains. The overall height of each stack represents the conservation of the sequence at that position. The relative position of amino acids was marked below. (PNG 236 kb)

Additional file 6: **Table S2** The distribution of GpWRKYs from *G. pentaphyllum*. (XLSX 10 kb)

Additional file 7: **Figure S5.** Sequence logo of GpbHLH domains. The overall height of each stack represents the conservation of the sequence at that position. The relative position of amino acids was marked below. (PNG 228 kb)

Additional file 8: **Table S3** The distribution of GpbHLHs from *G. pentaphyllum*. (XLSX 12 kb)

Additional file 9: **Figure S6.** Picture of *G. pentaphyllum*. (PNG 1314 kb)

Additional file 10: **Table S4.** The primers used for real-time PCR. (XLSX 14 kb)

## Data Availability

The sequence reads have been deposited in the NCBI database (https://www.ncbi.nlm.nih.gov) under the accession number PRJNA493176. In addition, the datasets supporting the conclusions of this article are included within the article and its additional files.

## Abbreviations

BAS: β-amyrin synthase
CAS: Cycloartenol synthase
CBC: Chair-boat-chair
CbQ: Cucurbitadienol synthase
CCC: Chair-chair-chair
CYP450s: Cytochrome P450
CYP716A47, PPDS: Protopanaxadiol synthase
CYP716A52v2: β-amyrin-28 oxidase
CYP716A53v2, PPTS: Protopanaxatriol synthase
DMAPP: Dimethylallyl pyrophosphate
DS: Dammarenediol-II synthase
FPP: Farnesyl pyrophosphate
FPS: Farnesyl pyrophosphate synthase
IPP: Isopentenyl diphosphate
Iso-Seq: Isoform sequencing
LS: Lanosterol synthase
LUS: Lupeol synthase
MeJA: Methyl jasmonate
MEP: Methylerythritol phosphate
MVA: Mevalonic acid
NGS: Next-generation sequencing
OSCs: 2,3-oxidosqualene cyclases
SE: Squalene epoxidase
SS: Squalene synthase
TFs: Transcription factors
UGTs: UDP-glycosyltransferase
