# Supplementary figures and images for: Hybrid sequencing of the Gynostemma pentaphyllum transcriptome provides new insights into gypenoside biosynthesis
Source: BMC Genomics. 2019 Aug 5;20:632. doi: 10.1186/s12864-019-6000-y (PMC6683540; doi:10.1186/s12864-019-6000-y)

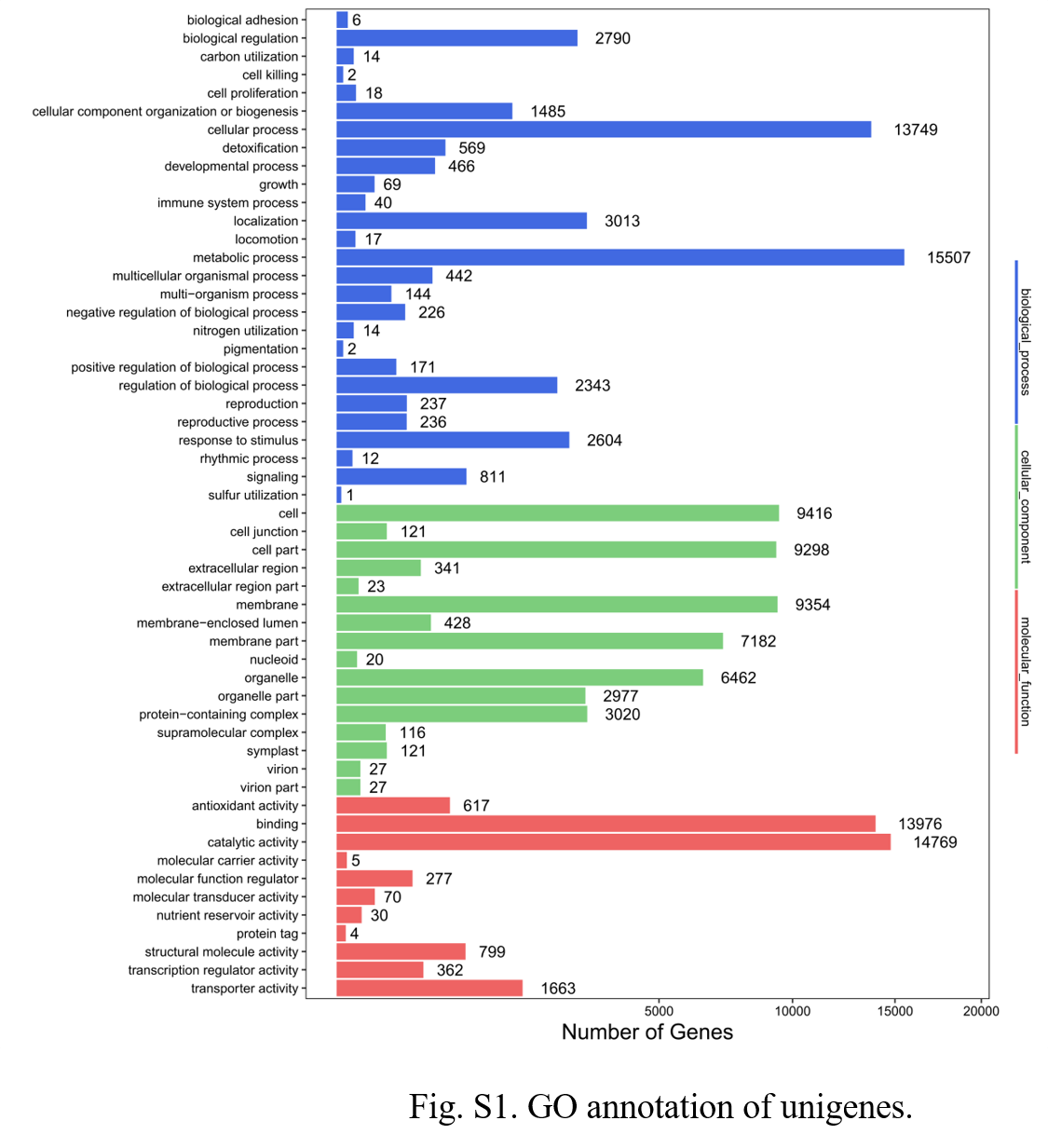

Supplement: Supplementary file 2 — Figure S1. GO annotation of unigenes. (PNG 200 kb) [file 12864_2019_6000_MOESM2_ESM.png]

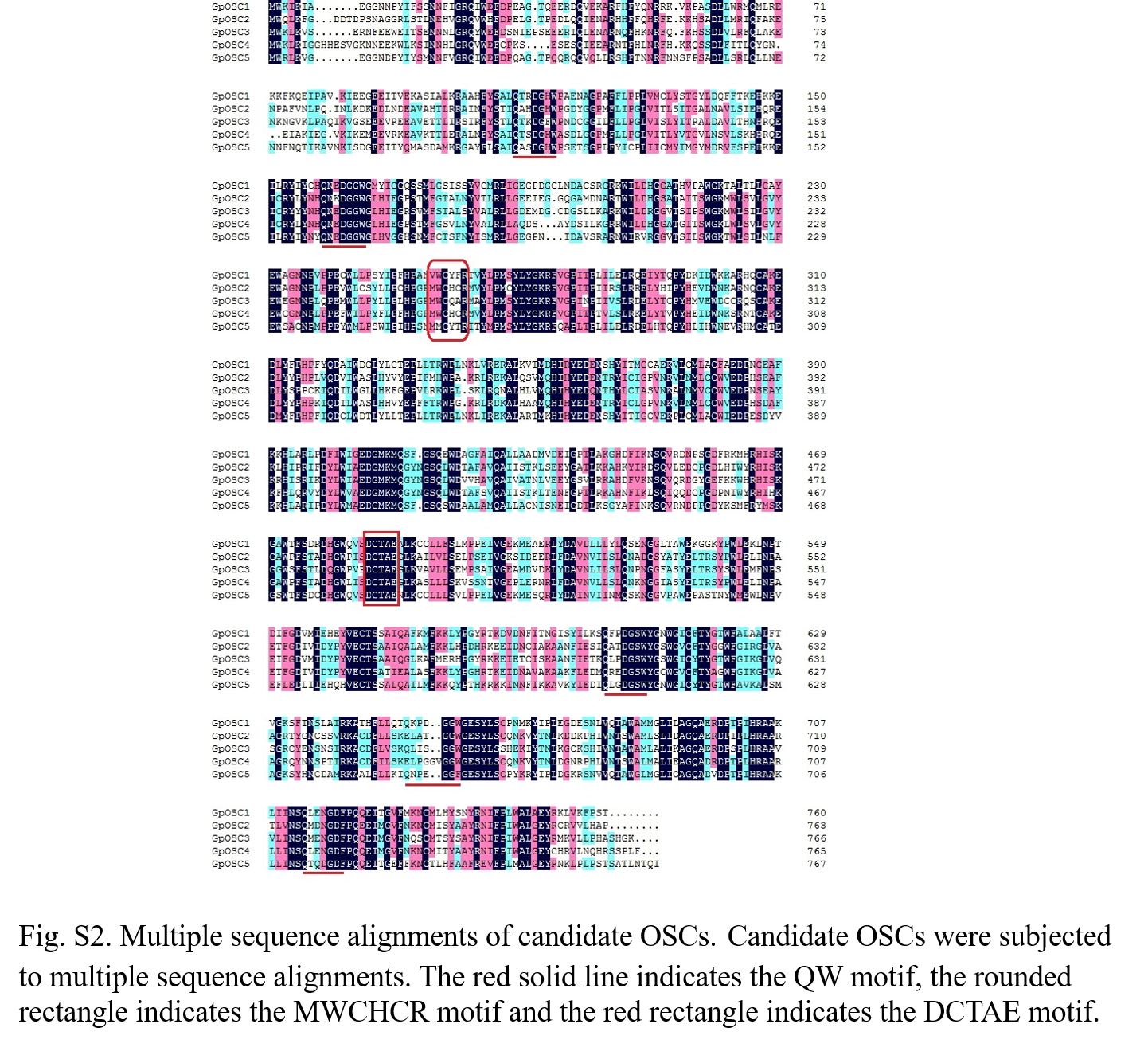

Supplement: Supplementary file 3 — Figure S2. Multiple sequence alignments of candidate OSCs. Candidate OSCs were subjected to multiple sequence alignments. The red solid line indicates the QW motif, the rounded rectangle indicates the MWCHCR motif and the red rectangle indicates the DCTAE motif. (PNG 1760 kb) [file 12864_2019_6000_MOESM3_ESM.png]

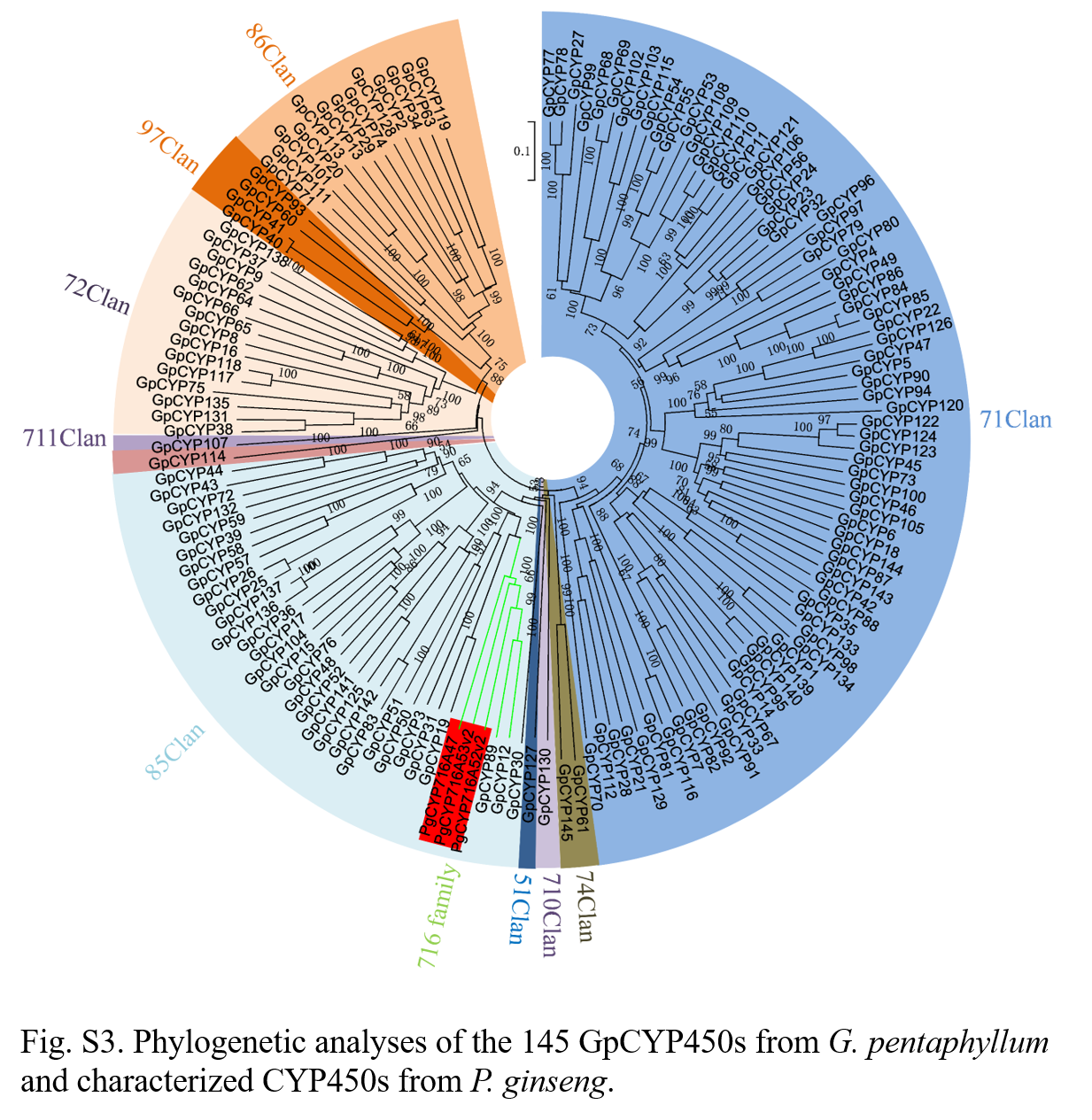

Supplement: Supplementary file 4 — Figure S3. Phylogenetic analyses of the 145 GpCYP450s from G. pentaphyllum and characterized CYP450s from P. ginseng. (PNG 928 kb) [file 12864_2019_6000_MOESM4_ESM.png]

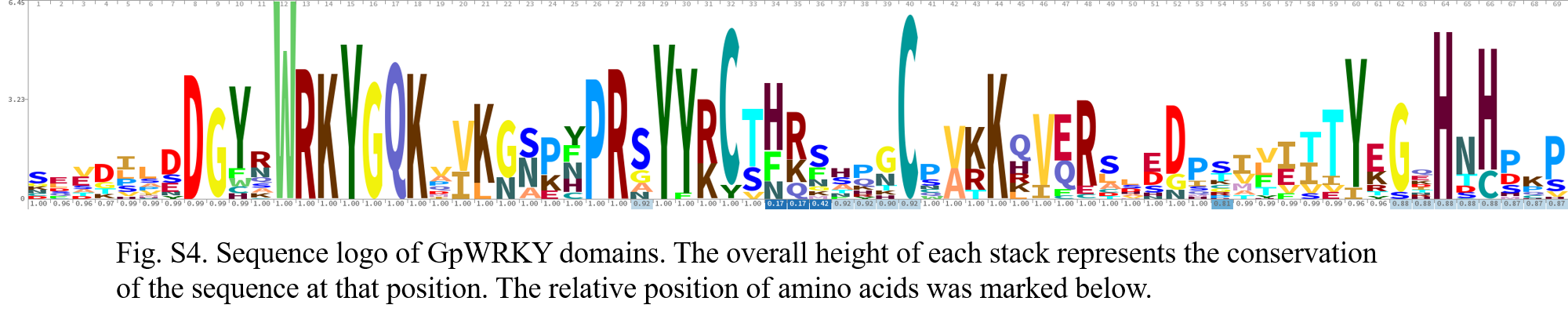

Supplement: Supplementary file 5 — Figure S4. Sequence logo of GpWRKY domains. The overall height of each stack represents the conservation of the sequence at that position. The relative position of amino acids was marked below. (PNG 236 kb) [file 12864_2019_6000_MOESM5_ESM.png]

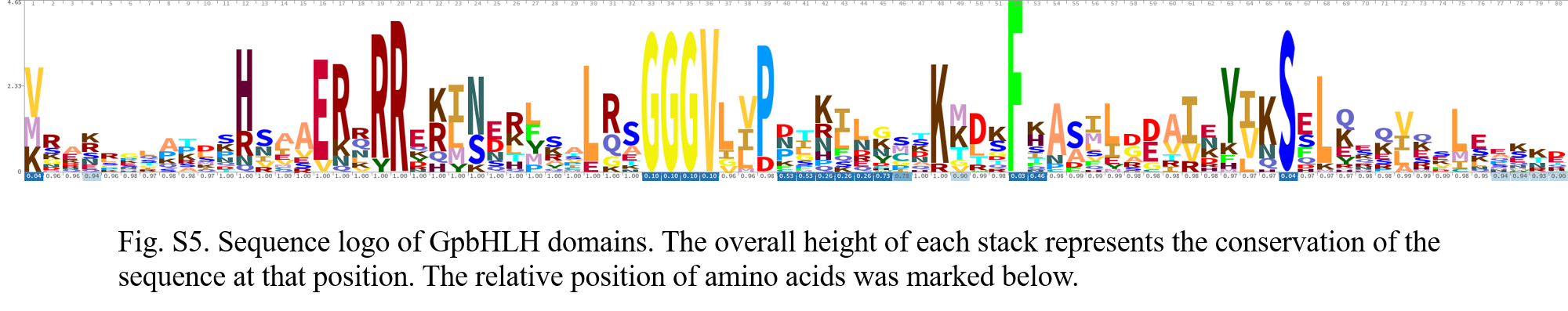

Supplement: Supplementary file 7 — Figure S5. Sequence logo of GpbHLH domains. The overall height of each stack represents the conservation of the sequence at that position. The relative position of amino acids was marked below. (PNG 228 kb) [file 12864_2019_6000_MOESM7_ESM.png]

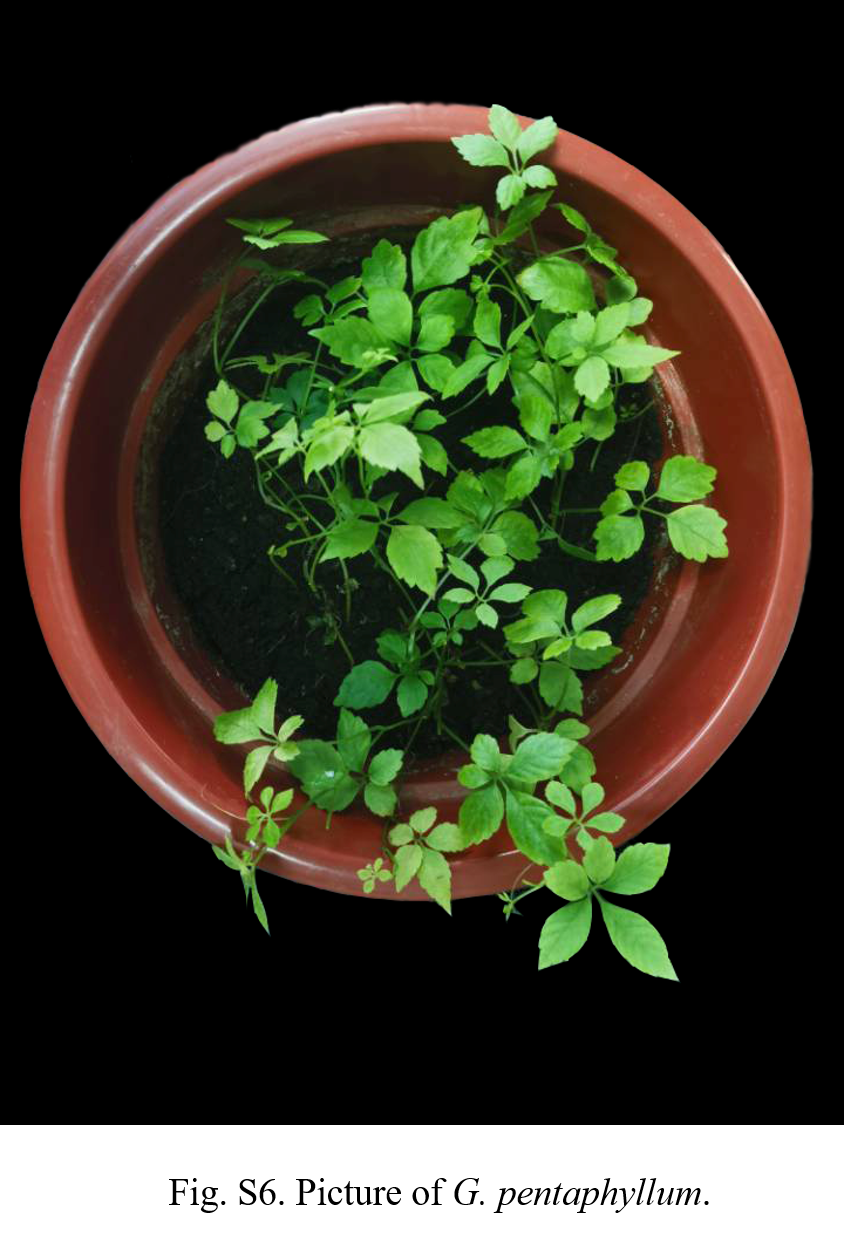

Supplement: Supplementary file 9 — Figure S6. Picture of G. pentaphyllum. (PNG 1314 kb) [file 12864_2019_6000_MOESM9_ESM.png]
